# Ethnopharmacological survey of six medicinal plants from Mali, West-Africa

**DOI:** 10.1186/1746-4269-4-26

**Published:** 2008-12-27

**Authors:** Tom Erik Grønhaug, Silje Glæserud, Mona Skogsrud, Ngolo Ballo, Sekou Bah, Drissa Diallo, Berit Smestad Paulsen

**Affiliations:** 1 Department of Pharmaceutical Chemistry, School of Pharmacy, University of Oslo, PO Box 1068 Blindern, 0316 Oslo, Norway; 2 Department of Traditional Medicine, BP 1746, Bamako, Mali

## Abstract

An ethnopharmacological survey was carried out to collect information about the use of six medicinal plants in the regions around Siby and Dioila, Mali. The plants investigated were *Biopyhtum petersianum, Cola cordifolia, Combretum molle, Opilia celtidifolia, Parkia biglobosa *and *Ximenia americana*.

More than 60 medical indications were reported for the use of these plants in traditional medicine. The most frequently reported ailments were malaria (25.6%), different types of pain (14.0%) and dermatitis (7.4%). The main forms for preparation were decoction (58.1%) and powdered plant material (28.4%). The most frequent used plant parts were leaves (37.7%) and stem bark (18.6%). The healers' consensus for the main indications is fairly high for the four plants *B. petersianum, C. cordifolia, C. molle *and *O. celtidifolia*, and this supports the traditional use of these plants. However for *P. biglobosa *and *X. americana *the healers' consensus is less consistent and it is more difficult to draw conclusions about the most important traditional use of these two plants.

## Background

Mali is a landlocked country located in West-Africa, with an area of approximately 1,246,000 km^2 ^for an estimated 13.5 million inhabitants. The country is composed of various climatic zones and a diversity of ethnic groups. The life expectancy in Mali is about 44 years for males and 47 years for females. This disturbing health situation of short life expectancy is due to the predominance of infectious diseases and parasites. To improve their health, people use both conventional and traditional medicine. In 2004 the density of physicians in Mali was 0.08 per 1000 inhabitants while already in 1978 there was one healer per 500 inhabitants in Mali [1,2].

Traditional medicine still remains the main resource for a large majority of people treating health problems. Being a comprehensive knowledge system, traditional medicine encompasses the utilization of substances, dosages and practices based on socio-cultural norms and religious beliefs as well as witnessed experiences and observations of a specific group. This knowledge is handed down from generation to generation in order to diagnose, prevent or eliminate a physical, social or spiritual imbalance [1].

In Mali, the Department of Traditional Medicine (DMT) is a collaborating centre of the World Health Organization (WHO) research for traditional medicine. The DMT has as one of their primary objectives to assure that traditional medicine is complementary to conventional medicine, assuming that the medicines can be produced from local resources, especially from medicinal plants [1]. DMT has carried out many phytochemical, pharmacological and toxicological studies with the ultimate goal of providing effective and non-toxic medicine to the population. There are several factors that are taken into account when selecting plants to be studied. One important factor is that several healers from different regions use the same plant to treat the same disease, and that the disease which the plant is used against represents a public health problem. From this work seven improved traditional medicines (ITMs) have been recognized as essential medicines in Mali and are being sold alongside conventional medicines in pharmacies [1].

The plants in our survey were chosen by DMT as they want to investigate if these plants are suitable candidates for development into new ITMs.

## Methodology

An ethnopharmacological survey was carried out in the regions around Siby and Dioila in November and December 2007, and a total of 58 healers were interviewed. 27 healers in the villages Siby, Dogoro and Gouena (Siby area) and 1 healer in the village Sirimabougou (Dioila area) were interviewed about the medicinal usage of *Biophytum petersianum *Klotzsch. (Oxalidaceae) and *Opilia celtidifolia *Endl. ex Walp. (Opiliaceae). 30 healers in the villages Banko, Sirimabougou and Ngolobougou (Dioila area) were interviewed about the medicinal usage of *Cola cordifolia *Sim (Sterculiaceae), *Combretum molle *R.Br. ex G.Don. (Combretaceae), *Parkia biglobosa *Benth. (Leguminosae) and *Ximenia americana *L. (Olacaceae).

The healers were asked whether or not they were using the plants in their practice. When a healer was using a plant in his practice, the information collected also included indication, part(s) of the plant being used, method(s) of preparation and details of administration, including the approximate amounts and number of doses per day. In some cases the healers reported the use of the *Loranthus spp. *when asked about a specific plant. Although the *Loranthus spp. *is distinct species it is discussed as a part of the plant it grows on because the healers see it as a part of the plant in question. The interviews were performed in the Bambara language with Professor Drissa Diallo and Doctor Sekou Bah, DMT, Bamako, and Ngolo Ballo, a plant systematist at DMT, as interpreters.

Prior to the interviews the healers were given information about the project, the participants in the survey and its goals. In respect of tradition, gifts of cola nuts (*Cola nitida *(Sterculiaceae)) and money were bestowed upon the traditional healers. The conversations with the healers are built on trust with the common goal of increasing the knowledge on medicinal plants and improving the health situation in the country.

## Results and discussion

### *Biophytum petersianum *Klotzsch. (Oxalidaceae)

= *Biophytum sensitivum *(L.) DC. (Oxalidaceae) and *Oxalis sensitiva *L. (Oxalidaceae)

Local name: Yeleni Nèloutogo and Djutogoui

*Biopyhtum petersianum *is a slender annual herb with stems up to 25 cm long; it has leaves in a terminal crown which are very sensitive. It is widespread in tropical and subtropical Africa, and across Asia to New Guinea [3].

#### Traditional use

**Figure 1:**
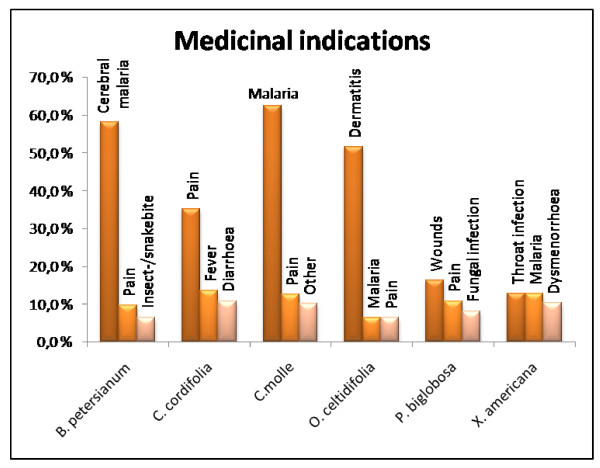
**Medicinal indications**. The three main medicinal indications for each plant.

**Figure 2:**
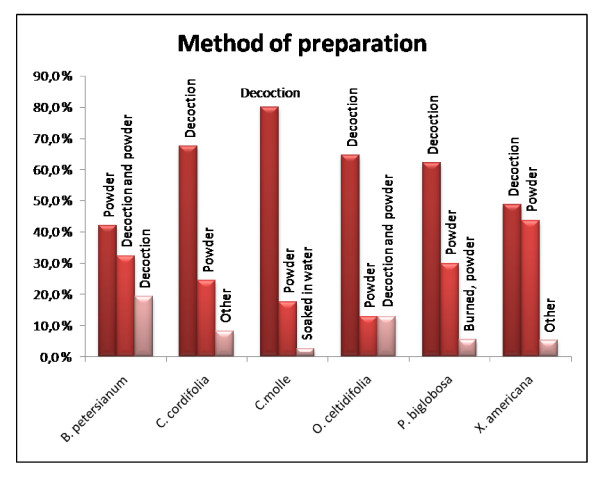
**Method of preparation**. The three main methods of preparation for each plant.

**Figure 3:**
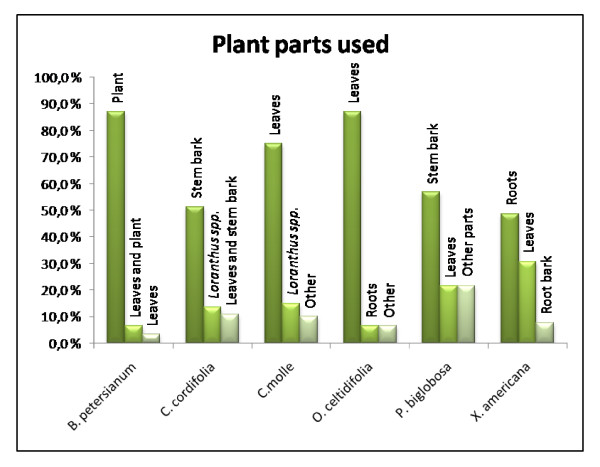
**Plant part used**. The three main plant parts used for each plant.

For *B. petersianum *the main indication in our survey was cerebral malaria (58.1% of the reported ailments for *B. petersianum*), while treatment of different types of pain was the second most cited ailment (9.7%) (Fig. 1). Various other ailments were also reported (Table 1, see additional file 1). A preparation of a powder from the plant was the most common preparation (41.9%) followed by a combination of powder and decoction (32.3%) and decoction (19.4%) (Fig. 2). The whole plant is the most frequent plant part used (Fig. 3). The healers' agreement about the main indication is fairly high and this supports the traditional use of *B. petersianum *against cerebral malaria.

According to the literature *B. petersianum *is used in Mali as a wound healing remedy [4,5] and against malaria and stomachache [4]. The plant is also used against stomachache in Nigeria, and in Gabon the roots and seeds are consider to be purgative. The powdered seed mixed with shea-butter is applied to wounds in Nigeria and a root decoction is used against gonorrhoea and kidney stone. In Cameroon and Mozambique the plant is used as an antidote against scorpion sting and snake bite [3].

Because of its highly sensitive leaves, which fold up in response to weather or touch, *B. petersianum *is used in magic and sorcery for several different purposes [3].

#### Biological activities

Methanolic extract has shown effect on COX-2 expression [6], and different types of polyphenolic compounds, some with effect on the COX-1/-2 system, have been isolated from *B. *sensitivum [7-10]. Different types of extracts from the plant have shown anti-inflammatory activity on the carrageenan-rat paw endema [11] and complement fixing polysaccharides from the plant have been reported [4,5]. The results above may explain the traditional use in treatment of malaria as the complement system is a part of the innate immune system and activating of the immune system can accelerate the body's immune response to malaria. The use in treatment of pain may be explained by the plant extract's modulation of COX expression and anti-inflammatory activity. Guruvayoorappan and Kuttan [12,13] have recently been performing a large scale investigation of antitumor effects of *Biophytum sensitivum*, and they have found that methanolic extracts of *B. sensitivum *have apoptotic effect on B16F-10 cells, and regulatory effects on NO- and cytokine production on tumor-associated macrophages. They also found that the methanolic extract stimulates the immune cell system in mice, leading to immune cell proliferation and that this, in turn, can stimulate NK cell-mediated tumor lysis.

### *Cola cordifolia *Sim (Sterculiaceae)

Local name: N'tabanokò

*Cola cordifolia *grows on the savannah in Senegal to Mali and is a large tree up to 15–25 m high, with a short buttressed trunk, low-branching with a dense crown [14].

#### Traditional use

*Cola cordifolia *is a remedy used to cure several diseases. In our survey different types of pain is the most frequent reported ailment (35.1% of the reported ailments for *C. cordifolia*) followed by fever (13.5%) and diarrhoea (10.8%) (Fig. 1). Various other ailments were also reported (Table 1, see additional file 1). The stem bark of *C. cordifolia *was used most frequently (51.4%) followed by the *Loranthus spp. *of the plant (13.5%) and the combination of leaves and stem bark (10.8%) (Fig. 3). The two most used preparations were decoction (67.6%) and preparation of a powder (24.3%) (Fig. 2). The decoction is usually used for a bath and/or to drink. The powder is usually dissolved in water and used for a bath and/or to drink, or the powder is thrown into fire and the smoke is inhaled. The healers' consensus for the main indication is fairly high, and this supports the traditional use of *C. cordifolia *as a remedy to treat different types of pain.

According to literature a decoction of the powdered bark of *C. cordifolia *is used in Mali for treatment of old wounds [5]. An ethnopharmacological survey carried out in the areas around Dioila and Bandiagara, Mali, indicated that the plant was used in treating a wide variety of ailments [15]. The bark is also used in Casamance, Senegal, in maceration for chest-affections, and together with other medicinal plants for blennorrhoea. A water-concoction is drunk in The Gambia for constipation, and the inner bark applied to a swollen finger is said to hasten maturation of pus [14].

Maceration of the twigs is administered in Casamance, Senegal, to facilitate childbirth. Water in which split-up small branches have been soaked is taken in The Gambia as a diuretic. A leaf-macerate is used in Senegal in leprosy treatment, and sap from leaf petioles is used in The Gambia in eye-treatments [14].

Root taken from the eastside of the tree, split up and put in a bottle with water is deemed helpful in The Gambia to get rid of gonorrhoea [14].

#### Biological activities

Despite the traditional medicinal use of the plant, few studies have been performed on the plant. In 1988 Diop et al. [16] investigated the Vitamin C content in fresh fruits from West-Africa. Togola et al. [15] found that the crude water extract had complement fixing activity and this could implement a role in activating the immune system. These results may explain the medicinal use since the activating of the immune system is involved in the immune response fighting diseases.

### *Combretum molle *R.Br. ex G.Don. (Combretaceae)

Local name: Ganianka

*Combretum molle *is a shrub or small tree to 10 m high, rarely 16 m, with a straight regular bole to 1 m girth, of savannah forest, from Senegal to West-Cameroon, and widespread in tropical Africa [17].

#### Traditional use

*Combretum molle *is used as a remedy to cure several diseases. In our survey the main indication is malaria (62.5% of the reported ailments for *C. molle*) followed by different types of pain (12.5%) (Fig. 1). Various other ailments were also reported (Table 1, see additional file 1). The most frequent part of the plant used is the leaves (75.0%) and the *Loranthus spp. *of the plant (15.0%) (Fig. 3). Decoction (80.0%) and a preparation of powder (17.5%) are the most commonly used preparations (Fig. 2). The decoction is usually used for a bath and/or to drink. The powder is usually suspended in water and used for a bath and/or to drink, or the powder is thrown into fire and the smoke is inhaled. The healers' agreement about the main indication is fairly high and this supports the traditional use of *C. molle *against malaria.

4 healers used the *Loranthus spp. *of *C. molle *for fortune seeking and to chase away evil spirits/and curses.

According to the literature *C. molle *is used in wound healing procedures in the Bamako region, Mali [5]. In Senegal the plant is held to be cholagogic, but inferior to *Combretum crotonoides *(Combretaceae) and *Combretum micranthum *(Combretaceae), and an aqueous suspension of powdered bark together with the mumuye gum is used as a gargle and in draught for sore-throat. The Dagarar of Burkina Faso applies powdered bark to sores. The bark along with cereal foods is taken for dysentery, and is used in ceremonial preparation for young children to prevent sickness and other troubles [17].

Leaves are prepared as a decoction for baths and draughts, or powdered and added to food in treatment of dropsy, ascites and oedemas in Senegal. In Ivory Coast and Nigeria this plant is used in the absence of that popular panacea, kinkeliba (*C. micranthum*) for treating jaundice and yellow fever, and in Burkina Faso it is given for abdominal complaints, diarrhoea etc., blennorrhoea, anuria, etc., and sometimes to women in childbirth to hasten the expulsion of the after-birth. Lobi of Burkina Faso often takes a decoction of leafy twigs in draughts and baths for bronchial affections and Bobo of Ivory Coast consider the plant a poison-antidote. The Maninka also treats whitlows by steeping the affected part in a leaf-decoction. Alcoholic extracts of leaves with water extracts of twigs have shown capacity to reduce sarcoma tumors in animals [17].

In the southern part of Senegal the plant is ascribed with magical properties by the Fula and Fouladou to promote courage in battle by inhaling smoke emitted from fire on which bark and branches have been placed. The wood is never used to stoke domestic hearths [17].

#### Biological activities

Some studies have been performed on the biological activities of *C. molle*. Ojewole [18,19] found analgesic, anti-inflammatory and cardiovascular effects of mollic acid glucoside isolated from *C. molle *leaves. Asres et al. [20] reported that the acetone extract of leaves from *C. molle *has antiprotozoal activity. In an investigation of the biological activity of different *Combretum spp. *McGaw et al. [21] found *C. molle *to have both anti-inflammatory and antischistosomal activity. These findings indicating that *C. molle *has antiprotozoal and anti-inflammatory activity may explain the traditional use of the plant against malaria and pain.

### *Opilia celtidifolia *Endl. ex Walp. (Opiliaceae)

Local name: Korôgué

*Opilia celtidifolia *is a woody climber, spreading, heavily-branched shrub or tree up to 10 m high, which grows in fringing forest and savannah, often on anthills. It is widespread from Senegal to Nigeria and dispersed over the drier parts of tropical Africa [3].

#### Traditional use

The main indication for *O. celtidifolia *in our survey was dermatitis (51.6% of the reported ailments for *O. celtidifolia*). The use of *O. celtidifolia *in treatment of dermatitis in Mali has also been previously reported by Togola et al. in 2005 [22], where an ethnopharmacological survey showed that dermatitis was the main indication. In Mali the term dermatitis is used as a common term for all kinds of skin disorders. Various other ailments were also reported, but only one or two healers reported usage of the plant for each ailment (Table 1, see additional file 1 and Fig. 1). The leaves of *O. celtidifolia *are the most frequent plant part used in traditional medicine (87.1% of the cases) (Fig. 3), and a decoction (64.5%) is the most common preparation (Fig. 2). The healers' consensus for the main indication is fairly high, and this supports the traditional use of *O. celtidifolia *as a remedy to treat dermatitis.

According to the literature the leaves of *O. celtidifolia *are mainly used to treat dermatitis and malaria in Mali [22], but the plant is also used in wound healing procedures [5,23]. A decoction of the leaves is used against fever in Ivory Coast. In Senegal a decoction is used as a gargle for dental abscesses, to treat oedema and as a general stimulant. The plant has internal and external use; against leprosy, acting as a purgative, and leaves reduced to ash are used against headache. The plant is said to have a violent action on the digestion system, and the stem and root are anthelmintic. In Nigeria the plant is primarily used in veterinary medicine [3].

#### Biological activities

In the literature there are only a few surveys on the biological activity of *O. celtidifolia*, most of the previous work have been focused on the chemistry of the plant. Togola et al. [24] isolated polysaccharides with complement fixing and macrophage stimulating activity from the water extract. Both the complement system and the macrophages are part of the immune system and play a role in the wound healing process. The activation of these processes may indicate that the polysaccharides from *O. celtidifolia *play a role in the wound healing process and this could explain the extensive use of different leaf preparations against dermatitis in Mali. In earlier work Shihata et al. [25] isolated saponins from the methanol extract and found anthelmintic and antispasmodic activities for these compounds. Saponins are reported to have a number of biological activities, among them immunostimulatory, antipyretic and antiparasitic effects [26]. The effects shown for saponins could explain the use of *O. celtidifolia *as a remedy for dermatitis, fever and internal worms.

### *Parkia biglobosa *Benth. (Leguminosae)

Local name: Nèrè

*Parkia biglobosa *is a tree being up to 20 m high, bole stout, not butteressed, low-branching, bearing a large wide-spreading crown, deciduous, flowering while leafless; flowers in pendulous capitula bearing also pendulous, large fruit pods; of the Soudanian/Guinean savannah and transition woodland, from Senegal across the Region and on into southern Sudan [27].

#### Traditional use

*Parkia biglobosa *is used as a remedy in our survey to treat different types of wounds (16.2% of the reported ailments for *P. biglobosa*) and pain (10.8%), and these are the most frequent reported ailments followed fungal infection (8.1%) (Fig. 1). *Parkia biglobosa *is also used against various other ailments (Table 1, see additional file 1). The stem bark of *P. biglobosa *was used most frequently (56.8%) followed by the leaves of the plant (21.6%) (Fig. 3). The two most used preparations were decoction (62.2%) and preparation of a powder (29.7%) (Fig. 2). The decoction is usually used for a bath and/or to drink. The powder is usually dissolved in water and used for a bath and/or to drink, or the powder is thrown into fire and the smoke is inhaled. The healers' agreement for the main indication for *P. biglobosa *is relatively low and it is therefore more difficult to pinpoint the most important traditional use of this plant.

2 healers used *P. biglobosa *for fortune seeking and to chase away evil spirits/and curses.

According to the literature the leaves, bark and pods are used to treat new and old wounds in Dogonland, Mali [23]. The bark-infusion is taken in Kordofanan in Ivory Coast, Burkina Faso and South-East Nigeria as a tonic and anti-diarrhoetic. The bark is commonly sold in herbalist shops in the western part of the Region for the analgesic action it confers in mouthwashes and steam-inhalations for toothache. A red colour is imparted to the mouth while the saponins in the bark contribute asepsis. In Casmance, Senegal, the bark has wide usage: alone for female sterility, baths and by draughts, and similarly administrated in prescription with other drug-plants for skin-infections and leprosy, and for blennorrhoea. Fula and Tukulor people of Senegal drink a decoction against *Schistosoma *infection. In Ivory Coast and Burkina Faso the pounded bark with lemon juice added is applied to sores and ulcers; a decoction is considered anti-rachitic, tonic and febrifugal; and it is one of 32 other plants in a complex prescription used in the Kaya region for leprosy. The bark is chewed by men in The Gambia for lack of virility; the cause will then, it is said, be voided on going to the latrine [27].

The leaves have some undefined medicinal use in Niger. In Nigeria and The Gambia root and leaf are pounded together in water to produce an eye-lotion. In Senegal medicinal use of the leaf is restricted to external applications. The leaves (and bark) are used in eye-lotion. The crushed leaf is made up into poultices, and to dab on the lips to dissipate 'fever spots'. They are made into steam inhalations in The Gambia for toothache. Lightly heated, then crushed, the leaflets are applied to burns [27].

#### Biological activities

Agunu et al. [28] reported that *P. biglobosa *showed anti-diarrhoeal properties in mice. Kouadio et al. [29] showed that the hexane extract from the bark of *P. biglobosa *had some analgesic and anti-inflammatory effects. Asuzu and Harvey [30] reported the methanol extract of *P. biglobosa *has shown significant protection against the neurotoxic, haemotoxic and cytotoxic effects of venoms of poisonous snakes. Studies have been done to identify the chemical constituents of the bark, Araujo et al. [31] found sterols and triterpenes in the petroleum ether extract and Tringali et al. [32] identified long-chain ester of *trans*-ferulic acid, a mixture of long-chain *cis*-ferulates and different kinds of catechins. Catechins and ferulates are antioxidants, and their antioxidant properties may be responsible for some of the medicinal effect seen for this plant.

### *Ximenia americana* L. (Olacaceae)

Local name: Ntonkè

*Ximenia americana *is a very variable shrubby tree up to 5 m high. It is often semi-parasitic, with strong thorns, or thornless, in savannah. The tree grows from Senegal to West Cameroon and is also widely dispersed in tropical Africa, America and Asia [3].

#### Traditional use

*Ximenia americana *is used as a remedy to cure several diseases. In our survey throat infection (12.8% of the reported ailments for *X. americana*) and malaria (12.8%) are the most frequent reported ailments followed by dysmenorrhoea (10.3%) (Table 1, see additional file 1 and Fig. 1). The roots (48.7%) and leaves (30.8%) are most frequently used in the treatment of the ailments (Fig. 3). The two most used preparations were decoction (48.7%) and preparation of a powder (43.6%) (Fig. 2). The decoction is usually used for a bath and/or to drink. The powder is usually dispersed in water and used for a bath and/or to drink, or the powder is thrown into fire and the smoke is inhaled. The healers' consensus is less consistent for *X. americana *and it is therefore more difficult to say which medical indication that is the most important one.

4 healers used the *Loranthus spp. *of *X. americana *for fortune seeking and to chase away evil spirits/and curses.

According to the literature *X. americana *is well known for its medicinal properties, and all parts of the tree are used. In Mali wounds are treated with a wash with a decoction of leaves or a combination of a wash with a decoction of bark and applying powdered bark is to the wounds [5]. Powder from bark is used on insect stings leading to boils [23]. The pulverised bark and roots are used in West Africa on epidermal troubles: on ulcers, craw-craw, ringworm, sores, etc. Powdered bark is deemed to be a good cleansing agent. Tenda in Casamance, Senegal, soak infected feet in boiled water with crushed bark and salt and bind them in cloth overnight, and then dead skin will be sloughed off. Root-bark is put into febrifugal medicines in Casamance, Senegal, and the plant is used against schistosomiasis in Ivory Coast, Burkina Faso and Nigeria [3].

The roots have internal uses in Casamance, Senegal, to treat guinea-worm infection, against leprosy and impotence, and in Nigeria for fever diarrhoea, jaundice, stomatitis and toothache. For swelling of the face, powdered root is inhaled in Senegal in mixture with *Maerua angolensis *(Capparaceae). In Tanganyika, Ivory Coast and Burkina Faso the root is used against fevers and diarrhoea. Root-powder massaged on the gums is used in Ivory Coast and Burkina Faso for stomatitis and toothache. The root compounded with the root of *Annona chrysophylla *(Annonaceae) has been used in Nigeria for sleeping-sickness [3].

#### Biological activities

Voss et al. [33,34] found the water extract of *X. americana *to have potent anticancer activity and that this effect was due to a type II ribosome-inactivating protein, riproximin. Asres et al. [35] reported that extract from the stem bark of *X. americana *had antiviral activity against human immunodeficiency virus type 1 (HIV-1) and type 2 (HIV-2). Kone et. al [36] found that extract of *X. americana *to have antibacterial effect. Omer and Elnima [37] tested extracts of different polarity and from different parts of the plant and found the methanolic and aqueous extracts to have antimicrobial effects. Diallo et al. [5] reported that water extract from *X. americana *have a complement fixing ability. The results showing that *X. americana *have complement fixing and antimicrobial effect could explain the plants traditional use against infections and malaria in Mali.

### Plant parts used and mode of preparation

**Figure 4:**
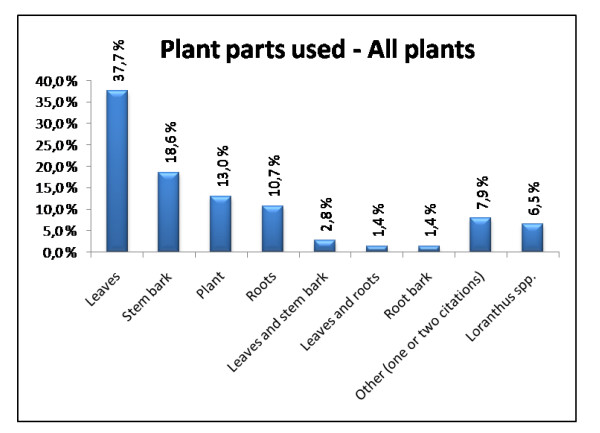
**Plant part used – All plants**. The most used plant parts overall.

Overall in our survey the leaves are the most frequently used plant part (37.7% of the citations for all plants), followed by the stem bark, the whole plant and the roots, 18.6%, 13.0% and 10.7% respectively. Preparations made of other plant parts and combinations of the parts mention above are more unusual, ranging from 0.5 – 2.8% (Fig. 4). The usages of the *Loranthus spp*. growing on the different medicinal plants were also reported quite frequently (6.5%) compared to earlier ethnopharmacological studies performed in Mali (usage not reported [22,23] and unpublished results).

In another ethnopharmacological study of medicinal plants in Dioila, Kolokani and Siby (Mali), Togola et al. [22] reported that the leaves (56.3%) and the roots (30.0%) were the most frequently used plant parts. The leaves (22%) and the roots (24%) were also the most frequently used parts in a survey performed in Dogonland (Mali) by Inngjerdingen et al. [23] in 2004. That the leaves are the most frequently used plant parts corresponds well with our own findings, but we found the stem bark to be the second most cited plant part used. The roots was only used 10.7% of the cases. There could be many reasons for this, but one explanation could be that the different surveys have been focused on different medicinal plants.

In our survey the overall results for used plant parts differ from the results of *C. cordifolia*, *P. biglobosa *and *X. americana*. The stem bark was the most frequent used plant part of *C. cordifolia *(51.4%) and *P. biglobosa *(56.8%). For *X. americana *the most frequent plant part used is the root (48.7%) (Fig. 3). Togola et al. [22] reported that the need and use of stem bark increased during the dry and windy season from February to May due to the lack of leaves during this period. Our survey was carried out in November-December after the rainy season so lack of leaves is probably not the explanation for the use of stem bark and roots in this case. A number of the healers also told us that they used stem bark from different sides of the tree (North, East, South and West). The reason for this could be to ensure that the tree will survive when they are collecting the bark, because removing larger areas of bark from just one side of the tree could expose so much of the trunk that the tree might acquire diseases and eventually dies.

**Figure 5:**
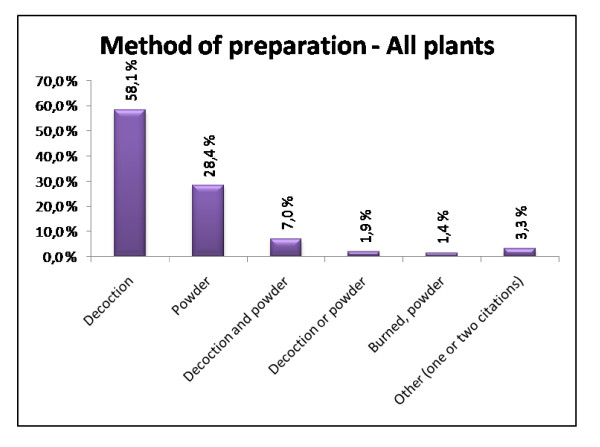
**Method of preparation – All plants**. The most used methods of preparation overall.

Decoction was by far the most used method of preparation (58.1% of the citations for all plants) followed by powder and a combination of decoction and powder (28.4% and 7.0% respectively). Other methods of preparations were also used, but only to a minor extent (Fig. 5). In another study performed by Togola et al. [22] decoction was also the most frequent way of preparation (65%) followed by infusion of powder (13%). These findings are similar to the results of our study.

### Administration and dosage

Oral and external applications as wash, steam bath or ointment are the administration forms that are most frequently used. Inhalation of steam was used in treatment of throat infections and fumigation was often used in the treatment of headache.

Different types of additives are sometimes used by the healers when preparing the remedies. Salt, food and drinks are used to improve taste of the preparation and thus the patients' compliance. Butter, oil and mud are used to make ointments or liniments for external use of the medicinal plants. The healers in our survey in some cases also used the combination of different medicinal plants to treat ailments (Table 1, see additional file 1).

For most ailments and remedies, the dosage given depends upon the sex, age, duration of illness, health and physical condition of the patient. The doses vary from 1 teacup for 1 day to 3–4 teacups 3 times a day for several days for oral administration. The same is noted when the treatment is a bath, often it is enough with a bath other times it is required several baths a day for a longer period of time. This variation was also noted when the plant was used to treat the same ailment. Togola et al. [22] suggested that the variation of the doses and duration of treatment from one healer to another may indicate that the plants have a low degree of toxicity. That corresponds well with our survey where the only side effects reported were diarrhoea and vomiting, and only in connection with *O. celtidifolia*.

## Conclusion

In the present study over 60 medicinal indications were reported for the six medicinal plants investigated. The healers' consensus for the main indications is fairly high for the four plants *B. petersianum, C. cordifolia, C. molle *and *O. celtidifolia*, and this supports the traditional use of these plants. However for *P. biglobosa *and *X. americana *the healers' consensus is less consistent and it is more difficult to draw conclusions about the most important traditional use of these two plants.

This survey complements the ongoing investigation of different medicinal plants from Mali. The ultimate goal for our project is to provide efficient and non-toxic medicines to the population in Mali, where medicinal plants still remains the main resource for a large majority of people treating health problems. Chemical characterization and the biological-, pharmacological- and toxicological activity of the plants are currently under investigation. Plant extract showing bioactivity corresponding to the traditional application of the plant can validate the traditional medicinal usage of the plant. These studies will hopefully provide important knowledge necessary for designing therapeutic agents from the plants.

## Competing interests

The authors declare that they have no competing interests.

## Authors' contributions

All authors performed the interviews with the healers and identified all plant material described.

TEG and BSP drafted and finalised the manuscript.

## Supplementary Material

Additional file 1 **Table 1**. Medical uses of six different plants in the regions of Siby and Dioila; Mali. The table shows the results from the interviews with the traditional healers. Click here for file
