# Supplementary material for: Ethnopharmacological survey of six medicinal plants from Mali, West-Africa
Source: J Ethnobiol Ethnomed. 2008 Dec 27;4:26. doi: 10.1186/1746-4269-4-26 (PMC2633328; doi:10.1186/1746-4269-4-26)
Supplement: Additional file 1 — Table 1. Medical uses of six different plants in the regions of Siby and Dioila; Mali. The table shows the results from the interviews with the traditional healers. [file 1746-4269-4-26-S1.doc]

**Table 1. Medical uses of six different plants in the regions of Siby and Dioila; Mali.**

Consensus, as percentages and number of citations, is written in parenthesis for the medicinal indications for each plant (including the *Loranthus spp.*).

| **Indications** | | **Plants parts used** | **Mode of preparation** | **Added substances** | **Mode of administration** |
| --- | --- | --- | --- | --- | --- |
| ***Biophytum petersianum* Klotzsch. (Oxalidaceae)** | | | | | |
| Cerebral malaria (58.1 % =18 ) | | Plant (15) | Powder (9) | Mixed with shea-butter / water / oil (8). | The mixture is applied onto the body. |
|  |  |  |  | Water and shea-butter (1) | Split into 2 parts. Infusion in water, body wash and mix with shea-butter, apply onto the body. |
|  |  |  | Decoction and powder (4) | Mixed with shea-butter / water / oil. | Oral and/or body bath and the mixture is applied onto the body. |
|  |  |  | Decoction (2) | *Combretum glutinosum* Hochst. ex A.Rich. (optionally). | Oral and body bath. |
|  |  | Leaves and Plant (2) | Decoction and powder | Shea-butter / oil. | Body bath. The mixture is applied onto the body. |
|  |  | Leaves | Decoction |  | Oral and body bath. If infants are ill, wash the mother’s breasts. |
| Pain (9.7 % = 3): | |  |  |  |  |
|  | -Pain when urinating | Plant | Decoction |  | Oral |
|  | -Stomachache (children) | Plant | Decoction |  | Oral |
|  | -Muscle pain | Unknown | - | - | - |
| Bite (6.5 % = 2): | |  |  |  |  |
|  | -Snakebite (wound healing) | Plant | Decoction |  | Oral, wash of bite |
|  | -Insect- / Snakebite | Plant | Powder | Oil | Wash of bite |

| Scared children / sleeplessness (6.5 % = 2) | | Plant | Decoction |  | Oral, eye wash |
| --- | --- | --- | --- | --- | --- |
| Aphrodisiac  (3.2 % =1) | | Plant | Powder | Salt and water (optionally). | Oral |
| Diarrhoea / weakness (3.2 % =1) | | Plant | Infusion |  | Oral |
| Increase appetite (3.2 % =1) | | Plant | Decoction |  | Oral and body wash |
| Measles (3.2 % =1) | | Plant | Powder |  | Oral and body wash |
| Teething (3.2 % =1) | | Plant | Powder | Water | Oral |
| Tonic (children) (3.2 % =1) | | Plant | Decoction |  | Oral and body wash (large amounts are needed). |
| ***Cola cordifolia* Sim. (Sterculiaceae)** | | | | | |
| Pain (35.1 % = 13): | |  |  |  |  |
|  | -Headache (6): |  |  |  |  |
|  | *Severe headache (4) | Leaves and *Loranthus spp.*(2) | Decoction Powder |  | Inhale steam, wash head and throw powder into fire, inhale smoke. |
|  |  | Leaves and stem bark | Decoction |  | Oral |
|  |  | Stem bark and *Loranthus spp.* | Powder |  | Thrown into fire, inhale smoke. |
|  | *Headache (2) | Stem bark (from N-, E-, S-, W-side of the tree) | Decoction | Sea salt (optionally) | Wash head or steam bath of head |
|  | -General pain (3) | Stem bark (from E- and W-side of the tree) | Decoction |  | Oral and/or body bath |
|  | -Chest pain (2) | Stem bark (from N-, E-, S-, W-side of the tree) | Decoction |  | Body bath and apply a piece of stem bark to the chest. |
|  |  | Branches | Burned | Butter from *Butyrospermum parkii* Kotschy. | Mixture of ash and butter is applied. |
|  | -Backache | Stem bark | Powder | Butter from *Butyrospermum parkii* Kotschy. | Mixture is massaged into the painful areas. |
|  | -Stomachache | Stem bark (from E- and W-side of the tree) | Decoction | Sea salt | Oral |
| Fever (adults and children) (13.5 % = 5) | | Stem bark (2) | Decoction |  | Oral and/or body bath |
|  |  | Leaves and stem bark (E-side of the tree) | Decoction |  | Oral and body bath |
|  |  | Leaves, roots and stem bark | Decoction |  | Oral and body bath |
|  |  | Leaves and stem bark | Decoction |  | Oral and body bath |
| Diarrhoea  (10.8 % = 4): | |  |  |  |  |
|  | -Diarrhoea (and coughing ) (3) | Stem bark (2) | Decoction |  | Oral and body bath |
|  |  | Leaves and stem bark | Decoction |  | Oral and body bath |
|  | -Diarrhoea (children) | Leaves | Decoction |  | Oral and body bath |
| Malaria (2.7 % = 1) | | Stem bark | Decoction |  | Body bath |
| Old wounds  (2.7 % = 1) | | Stem bark | 2 parts: 1) soaked in water 2) burned |  | Wound is washed with water and then covered with ash. |
| Asthma (2.7 % = 1) | | Leaves | Soaked in water for 7 days |  | Oral and body bath |
| Prevention of death of children  (2.7 % = 1) | | Stem bark | Decoction | (Is used to prevent that another child dies if a child have died when it was carried at the mothers back). | Body wash |
| Prevention of death of premature children (2.7 % = 1) | | Stem bark | Powder | Stem bark from the *Baba* tree and water. Food. | Body wash, consume |
| Low semen production  (2.7 % = 1) | | Stem bark (E- and W-side of tree) | Powder | Sea salt and water. | Oral |
| Oedema (2.7 % = 1) | | Stem bark | Powder | Water, patient should not eat sugar, salt or milk during the treatment. | Oral |
| Schistosomiasis  (2.7 % = 1) | | Roots (E- and W-side of tree) | Decoction |  | Oral and body wash |
| Swelling of the testis (2.7 % = 1) | | Leaves | Decoction | Leaves from an unknown plant. | Steam bath of the testis |
| Quicker recovery after childbirth  (2.7 % = 1) | | Stem bark (E- and W-side of tree) | Decoction |  | Steam bath, oral and body bath. |
| ***Loranthus spp.* of *Cola cordifolia*** | | | | | |
| Weight loss and malnutrition or patient sleeps a lot  (5.4 % = 2) | | *Loranthus spp.* | Decoction |  | Oral or steam bath and body bath |
| Cerebral malaria  (2.7 % = 1) | | *Loranthus spp.* | Powder | Water | Oral and body bath. |
| Old wounds  (2.7 % = 1) | | *Loranthus spp.* | Powder | Mud. | The wound is washed and the mixture is applied onto the wound. |
| Female infertility  (2.7 % = 1) | | *Loranthus spp.* | Powder | Drink | Oral |
| ***Combretum molle* R.Br. ex G.Don. (Combretaceae)** | | | | | |
| Malaria  (62.5 % = 25): | |  |  |  |  |
|  | Malaria (23) | Leaves | Decoction (18) |  | Oral and/or body bath and/or steam bath |
|  |  |  | Decoction (2) | Leaves from *Nauclea latifolia* Blanco. (optionally). | Oral and body bath |
|  |  |  | Decoction | Leaves from *Securinega virosa* (Roxb. ex Willd.) Baill. | Oral and body bath |
|  |  |  | Decoction | 4 sliced lemons. | Oral |
|  |  |  | Decoction | Leaves from *Combretum micranthum* G.Don. and stem bark from *Khaya senegalensis* A.Juss. | Oral |
|  | Malaria in children (2) | Leaves | Decoction |  | Oral and body bath |
|  |  | Leaves and stem bark | Decoction |  | Oral |
| Pain (12.5 % = 5): | |  |  |  |  |
|  | Chest pain | Leaves | Decoction |  | Oral and body bath |
|  | Chest pain in malaria | Leaves | Decoction |  | Oral and body bath |
|  | Generalized pain | Leaves | Decoction |  | Steam bath, oral and body bath |
|  | Headache | Leaves | Decoction |  | Steam bath and body bath. |
|  | Stomachache | Leaves | Powder | Root from *Securinega virosa* (Roxb. ex Willd.) Baill. | Oral |
| Impotence  (2.5 % = 1) | | Root bark and root | Powder | Sea salt, drink. | Oral |
| Madness / Aggressive behavior  (2.5 % = 1) | | Leaves and roots | Decoction |  | Oral and body bath |
| Schistosomiasis  (2.5 % = 1) | | Root | Soaked in water |  | Oral |
| Vomiting (2.5 % = 1) | | Leaves | Decoction |  | Oral. If the patient has joint pains, body bath. |
| ***Loranthus spp.* of *Combretum molle*** | | | | | |
| Headache  (7.5 % = 3) | | *Loranthus spp.* | Powder |  | The powder is thrown into the fire before bedtime and the smoke is inhaled |
| Malaria (2.5 % = 1) | | *Loranthus spp.* | Decoction |  | Oral and body bath |
| Infertility (2.5 % = 1) | | *Loranthus spp.* | Powder | Water (optionally) | The powder is either thrown into the fire and the smoke inhaled or used in body bath. |
| Nightmare  (2.5 % = 1) | | *Loranthus spp.* | Powder | Water (optionally) | The powder is either thrown into the fire and the smoke inhaled or used in body bath. |
| ***Opilia celtidifolia* Endl. ex Walp (Opiliaceae)** | | | | | |
| Dermatitis:  (51.6 % = 16) | |  |  |  |  |
|  | -Dermatitis (10) | Leaves | Decoction (7) |  | Body wash |
|  |  |  | Decoction | Roots / leaves / flowers from an unknown plant | Oral and body bath |
|  |  |  | Powder (2) | Oil | The mixture is applied onto the body. |
|  | -Dermatitis with small wounds (6) | Leaves | Decoction (3) |  | Body wash (and oral) |
|  |  |  | Decoction and powder (3) |  | Body wash (and oral) and apply powder to wounds. |
| Flatulence  (6.5 % = 2) | | Roots | Powder | Water | Oral. Side effect: Diarrhoea. |
|  | | Leaves and roots | Decoction or powder | Food. | Oral and body wash or consume. Side effect: Diarrhoea and vomiting. |
| Internal worm  (6.5 % = 2) | | Plant | Decoction |  | Oral. Overdose => Diarrhoea |
|  | | Leaves | Decoction or powder | Milk or bananas | Oral |
| Malaria (6.5 % = 2) | | Leaves | Decoction |  | Oral |
| Pain (6.5 % = 2): | |  |  |  |  |
|  | -Muscle pain | Leaves | Decoction |  | Body wash |
|  | -Stomachache / constipation | Roots | Powder |  | Oral |
| Anorexia (3.2 % = 1) | | Leaves | Decoction |  | Oral and body wash |
| Fever (3.2 % = 1) | | Leaves | Decoction |  | Body wash. Do not drink => side effect: Diarrhoea. |
| Icterus (3.2 % = 1) | | Leaves | Decoction |  | Oral |
| Oedema (3.2 % = 1) | | Leaves | Decoction or powder | Water | Steam bath and body bath or body bath |
| Rheumatism  (3.2 % = 1) | | Leaves | Decoction |  | -Body wash with a traditional soap before body wash with decoction. |
| Tonic (3.2 % = 1) | | Leaves | Decoction |  | Oral and body wash. |
| Wound healing  (3.2 % = 1) | | Leaves | Decoction and powder |  | Body wash and apply powder to wounds. |
| ***Parkia biglobosa* Benth. (Leguminosae)** | | | | | |
| Wounds  (16.2 % = 6): | |  |  |  |  |
|  | -Gastric ulcer (2) | Stem bark | Powder | Water (optionally) | Oral |
|  | -Wound healing (2) | Stem bark | Decoction |  | Wash the wounds |
|  | -Internal wounds | Stem bark | Powder |  | Oral |
|  | -Cold sore | Unknown | - | - | - |
| Pain (10.8 % = 4): | |  |  |  |  |
|  | Backache (2) | Leaves | Decoction |  | Steam bath of sore area. |
|  |  | Stem bark | Decoction | Root of *Entada africana* Guill. & Perr. | Oral and body bath. |
|  | Chest pain (children) | Leaves | Decoction |  | Oral and body bath. |
|  | Muscle pain | Leaves | Decoction |  | Oral and massage onto the sore area. |
| Fungal infection  (8.1 % = 3) | | Stem bark (E- and W-side from tree) | Decoction | Stem bark from *Butyrospermum parkii* Kotschy. (E- and W-side from both trees) (Optionally) | Oral or Oral and wash of anus or Oral and wash of infected area. |
| Throat infection  (5.4 % = 2) | | Leaves | Decoction | Sea salt | Sea salt is placed on the back of the tongue. Steam bath of mouth. |
|  |  |  | Powder | Water | Gargle |
| Liver infection  (5.4 % = 2) | | Stem bark (and root) | Decoction |  | Oral and body bath |
| Malaria (5.4 % = 2) | | Fruit | Powder | Water | A paste is made |
|  |  | Stem bark | Decoction |  | Oral and body bath |
| Polymenorrhea  (5.4 % = 2) | | Sap | Burned, powder |  | Oral |
|  |  | Stem bark (from E- and W-side of tree) | Decoction | White cola nut. | Oral |
| Amenorrhea  (2.7 % = 1) | | Stem bark and old fruits | Decoction |  | Oral and body bath |
| Caries (2.7 % = 1) | | Stem bark | Decoction |  | Mouthwash |
| Chronic constipation  (2.7 % = 1) | | Stem bark | Decoction | 3-4 pieces of lemon (squeezed) | Oral |
| Cough / short of breath after work  (2.7 % = 1) | | Stem bark | Powder | Stem bark from *Butyrospermum parkii* Kotschy. Sea salt, water. | Oral |
| Dysentery  (2.7 % = 1) | | Stem bark | Decoction | Sea salt. | Oral |
| Headache  (2.7 % = 1) | | Stem bark | Powder |  | Powder is thrown into fire and smoke inhaled. |
| Hemorrhoids  (2.7 % = 1) | | Stem bark | Powder | Hot water | Drink 1 part and fill en enema with the other part. |
| Hiccups (2.7 % = 1) | | Stem bark | Powder |  | Oral |
| Malnutrition (children)  (2.7 % = 1) | | Stem bark | Decoction |  | Body wash |
| Prevent epidemic (children)  (2.7 % = 1) | | Leaves | Decoction |  | Oral and body wash |
| Prophylaxis against infection / diseases (2.7 % = 1) | | Stem bark | Decoction |  | Body bath |
| Protection against airborne diseases (children)  (2.7 % = 1) | | Leaves (from a young tree) | Decoction |  | Body bath |
| Sexual infection  (2.7 % = 1) | | Stem bark | Powder | Stem bark from *Alternanthera repens* (L.) Link. Water and butter (optionally). | Oral and genital wash. If itch mix some powder with butter and apply. |
| Swelling of genitals (2.7 % = 1) | | Sap | Burned, powder | Butter from *Butyrospermum parkii* Kotschy. | The mixture is applied to the swollen parts. |
| Unknown disease (2.7 % = 1) | | Leaves | Decoction |  | Steam bath and body bath |
| ***Loranthus spp.* of *Parkia biglobosa*** | | | | | |
| Gastric ulcer  (2.7 % = 1) | | *Loranthus spp.* | Powder | Root from *Lannea microcarpa* Engl. & K.Krause. Water. | Oral |
| ***Ximenia americana* L. (Olacaceae)** | | | | | |
| Throat infection (12.8 % = 5) | | Leaves | Decoction | Sea salt (optionally), Chilli-powder (optionally). | Sea salt and chilli are placed on the back of the tongue. Steam bath of mouth (and oral) |
| Malaria  (12.8 % = 5): | |  |  |  |  |
|  | -Malaria (2) | Root bark | Powder |  | Oral |
|  |  | Root | Powder |  | Oral |
|  | -Cerebral malaria (3) | Leaves | Decoction | Leaves from *Sida spp.* | Oral and body bath |
|  |  | Leaves and stem bark | Decoction |  | Wash head and oral |
|  |  | Leaves and roots | Decoction |  | Steam bath and oral |
| Dysmenorrhoea (10.3 % = 4) | | Roots | Powder (2) | Water | Oral |
|  |  |  |  | Fruit from *Aframomum melegueta* K.Schum. Sea salt. | Add to drink |
|  |  |  | Decoction (2) | Roots from *Bridelia ferruginea* Benth.*, Cassia sieberiana* DC.*, Securidaca longepedunculata* Fresen. and *Securinega virosa* (Roxb. ex Willd.) Baill. Sea salt and rice. | Oral  Oral and body bath |
|  |  |  |  |  |  |
| Anxiety (7.8 % = 3) | |  |  |  |  |
|  | -Anxiety / nervousness (2) | Leaves | Decoction | Sea salt | Oral or sea salt is placed on the back of the tongue. Inhale steam |
|  | -Nightmare / anxiety in children | Root or the *Loranthus spp.* | Powder |  | Body bath |
| Caries (5.1 % = 2) | | Leaves | Decoction |  | Steam bath of mouth and mouth wash. |
| Schistosomiasis | | Root | Powder | Water | Oral |
|  | (5.1 % = 2) | Root bark | Powder | Water | Oral |
| Stomachache | | Root | Decoction |  | Oral |
|  | (5.1 % = 2) |  | Powder | Water | Oral |
| Amenorrhoea  (5.1 % = 2) | | Root | Mashed, divided into 2. Sundry one part – decoction. Shadow-dry one part - powder | Part 2 in drink | Oral |
| Constipation  (2.6 % = 1) | | Root | Powder | Water | Oral |
| Diarrhoea  (2.6 % = 1) | | Root | Powder | Cultured milk. | Oral |
| Dysentery  (2.6 % = 1) | | Root | Powder | Drink | Oral |
| Eye infection  (2.6 % = 1) | | Leaves | Decoction |  | Face wash |
| Flatulence  (2.6 % = 1) | | Root bark | Powder | Sea salt | Oral |
| Gastric ulcer  (2.6 % = 1) | | Root | Powder |  | Oral |
| Genital-itching  (2.6 % = 1) | | Root | Powder | Fruit from *Aframomum melegueta* K.Schum*.* Sea salt. | Oral |
| Hemorrhoids  (2.6 % = 1) | | Root | Burned, ash | Butter | 1 part oral and 1 part is mixed with butter and applied on/around rectum. |
| Hiccups  (2.6 % = 1) | | Leaves | Decoction |  | Inhalation of steam and oral |
| Incontinence  (2.6 % = 1) | | Root | Powder | Fruit from *Aframomum melegueta* K.Schum. Sea salt. | Oral |
| Female infertility  (2.6 % = 1) | | Root | Decoction |  | Oral |
| Liver infection  (2.6 % = 1) | | Root | Powder |  | Oral |
| Swelling of head and legs (2.6 % = 1) | | Root | Sundried, powder | Butter from *Butyrospermum parkii* Kotschy. | Mixture is massaged onto the swollen areas. If wounds, cover the wounds with mixture. |
| ***Loranthus spp.* of *Ximenia americana*** | | | | | |
| Night fever  (2.6 % = 1) | | *Loranthus spp.* | 2 ways: 1) Powder 2) Decoction |  | 1) Powder in fire, fill room with smoke and inhale smoke. 2) Oral and body bath. |
| Unknown disease  (2.6 % = 1) | | *Loranthus spp.* | Decoction | The *Loranthus spp.* of *Cola cordifolia*. | Body bath |
